# Supplementary material for: Identifying COVID-19 cases in outpatient settings
Source: Epidemiol Infect. 2021 Apr 5;149:e92. doi: 10.1017/S0950268821000704 (PMC8060539; doi:10.1017/S0950268821000704)
Supplement: Supplementary file 1 [file S0950268821000704sup001.docx]

**Supplementary materials**

1. *Mathematical notation*

Denote response variable as $Y$ with $n$ values representing subjects are control (0) or COVID-19 case (1), and covariates $X_{1}$, $X_{2}$, …, $X_{48}$ with 0/1 values indicating subjects having one of the 12 symptoms (37.5-37.9°C, ≥38.0°C, abdominal pain, cough, diarrhea, feverishness, headache, muscle ache, nausea or vomiting, runny nose, shortness of breath, sore throat) on one of the 4 illness days strata (1–2, 3–4, 5–7 and 8 or more since onset of the first symptom). For example, 37.5-37.9°C on day 1-2, cough on day 3-4, diarrhea on day 5-7, etc.

To remove collinearity of variables, generalised logistic regression with LASSO was used to filter out 27 variables, which were redefined as $X_{1}$, $X_{2}$, …, $X_{27}$: having 37.5-37.9°C on day 1-2, 37.5-37.9°C on day 3-4, ≥38.0°C on day 1-2, ≥38.0°C on day 3-4, ≥38.0°C on day 5-7, ≥38.0°C on day 8+, abdominal pain on day 1-2, cough on day 1-2, cough on day 3-4, diarrhea on day 1-2, diarrhea on day 3-4, diarrhea on day 5-7, diarrhea on day 8+, feverishness on day 1-2, feverishness on day 3-4, feverishness on day 5-7, feverishness on day 8+, headache on day 3-4, headache on day 5-7, muscle ache on day 1-2, runny nose on day 1-2, runny nose on day 3-4, runny nose on day 5-7, runny nose on day 8+, shortness of breath on day 8+, sore throat on day 1-2, sore throat on day 3-4.

The formula to model the probability $q$of having COVID-19 with symptoms of dynamic occurrence is expressed as

$\text{logit}(q)= \sum_{p=1}^{27} \beta_{p}X_{p}$,

where logit is the transformation function $\text{logit}\left( q \right)=\text{log}(\frac{q}{1-q})$, and $\beta_{p}$ denotes the coefficient of $X_{p}$.

1. *Weighting of cases*

We assume linearly decreasing number of cases until day 15, by which time all patients would have been admitted.

$$w(d)\propto\frac{1-d/15}{n(d)},$$

where $d=1,2,\cdots,14$ represents illness day and $n$ is the count of records in each respective day. To justify the necessity of decreasing trend in weightage, all COVID-19 patients are admitted to hospital or isolated in community care facility in our cohort in Singapore, therefore cases number decreases by days of illness in outpatient care setting. The calculated weights are incorporated into the generalized logistic regression model, which is used to analyze the predictive risks of symptom characteristics.

1. *Stochastic search algorithm*

To determine optimal multiple cutoffs across illness days with a minimum threshold of overall specificity, first, choose a cutoff range for each illness day group, 1–2, 3–4, 5–7 and 8 or more, initializing on 0.5-1 for all groups; second, randomly draw 1000 cutoffs for each of the illness day ranges; third, for each cutoff calculate the overall specificity and sensitivity from the predicted risks and obtaining the cutoff combination which maximized sensitivity. This process was repeated with 4 or 5 times, each time ranges of search narrowed in the first step, until satisfactory accuracy level is achieved, e.g., first decimal place, and we have the optimal cutoff combination according to specificity and sensitivity.
